# Mesenchymal stem cell transplantation for vaginal repair in an ovariectomized rhesus macaque model

**DOI:** 10.1186/s13287-021-02488-2

**Published:** 2021-07-15

**Authors:** Ye Zhang, Yidi Ma, Juan Chen, Min Wang, Yuan Cao, Lei Li, Hua Yang, Xudong Liu, Yaqian Li, Lan Zhu

**Affiliations:** 1 Department of Obstetrics and Gynecology, National Clinical Research Center for Obstetric & Gynecologic Diseases, State Key Laboratory of Complex Severe and Rare Diseases, Peking Union Medical College Hospital, Chinese Academy of Medical Sciences and Peking Union Medical College, Beijing, China; 2 Medical Science Research Center, State Key Laboratory of Complex Severe and Rare Diseases, Peking Union Medical College Hospital, Chinese Academy of Medical Sciences and Peking Union Medical College, Beijing, China; 3 Department of Rheumatology, Beijing Hospital, Chinese Academy of Medical Sciences and Peking Union Medical College, Beijing, China; 4 Department of Neurology, Peking Union Medical College Hospital, Chinese Academy of Medical Sciences and Peking Union Medical College, Beijing, China

**Keywords:** Human umbilical cord mesenchymal stem cell, Pelvic organ prolapse, Rhesus macaque model, Vaginal repair

## Abstract

**Background:**

Current surgical therapies for pelvic organ prolapse (POP) do not repair weak vaginal tissue and just provide support; these therapies may trigger severe complications. Stem cell-based regenerative therapy, due to its ability to reconstruct damaged tissue, may be a promising therapeutic strategy for POP. The objective of this study is to evaluate whether mesenchymal stem cell (MSC) therapy can repair weak vaginal tissue in an ovariectomized rhesus macaque model.

**Methods:**

A bilateral ovariectomy model was established in rhesus macaques to induce menopause-related vaginal injury. Ten bilaterally ovariectomized rhesus macaques were divided into two groups (n=5/group): the saline group and the MSC group. Three months after ovariectomy, saline or MSCs were injected in situ into the injured vaginal wall. The vaginal tissue was harvested 12 weeks after injection for histological and biochemical analyses to evaluate changes of extracellular matrix, microvascular density, and smooth muscle in the vaginal tissue. Biomechanical properties of the vaginal tissue were assessed by uniaxial tensile testing. Data analysis was performed with unpaired Student’s t test or Mann-Whitney.

**Results:**

Twelve weeks after MSC transplantation, histological and biochemical analyses revealed that the content of collagen I, elastin, and microvascular density in the lamina propria of the vagina increased significantly in the MSC group compared with the saline group. And the fraction of smooth muscle in the muscularis of vagina increased significantly in the MSC group. In addition, MSC transplantation improved the biomechanical properties of the vagina by enhancing the elastic modulus.

**Conclusion:**

Vaginal MSC transplantation could repair the weak vaginal tissue by promoting extracellular matrix ingrowth, neovascularization, and smooth muscle formation and improve the biomechanical properties of the vagina, providing a new prospective treatment for POP.

**Supplementary Information:**

The online version contains supplementary material available at 10.1186/s13287-021-02488-2.

## Background

Pelvic organ prolapse (POP) is defined as the descent of the anterior or posterior vaginal wall, the uterus, or the apex of the vagina after hysterectomy [1]. Although POP is a nonfatal disease, it seriously affects a woman’s quality of life by its local physical symptoms of urinary incontinence, voiding difficulty, anal incontinence, and sexual dysfunction. The prevalence of POP is 9.6–30.8% in women, particularly in postmenopausal women [2, 3]. The lifetime risk of undergoing surgery for POP is 11–19% [4, 5], which places a major economic burden on patients and the healthcare system [6]. Surgical strategies for POP repair mainly include native tissue repair and mesh-augmented repair strategies. Native tissue repair has high objective failure rates, and although mesh repair has a reduced failure rate, postoperative complications such as infection, chronic pain, and vaginal erosion have caused international controversies, limiting its use [7, 8]. FDA warnings regarding these adverse complications have led to the ban of several transvaginal meshes in many countries. Therefore, the development of a novel therapeutic strategy for POP with a high cure rate and few complications is needed.

The exact pathophysiology of POP has not been well characterized. Vaginal delivery, menopause, and connective tissue abnormalities predispose some women to disruption, stretching, and dysfunction of the vagina, resulting in POP [9–12]. Previous studies have shown collagen disequilibrium and a reduced amount of smooth muscle in the vaginal tissue of POP patients [13–15]. However, current clinical therapies for POP do not treat these pathophysiological causes and focus only on the recovery of anatomical positioning. Therefore, repair and restoration of the vaginal tissue composition should be seriously considered in the development of novel treatments for POP.

Stem cell therapies have been used in many medical areas to replace, repair, or enhance the biological function of damaged tissue or organs, such as in skin regeneration, trachea reconstruction, joint replacement, and bladder repair [16]. Among the different existing stem cell populations, mesenchymal stem cells (MSCs) have gathered attention and emerged as attractive candidates for various therapeutic applications due to their characteristics, including their multilineage differentiation potential and ability to exert paracrine effects. Several scientific studies have been performed on stem cell-based therapies for POP [17–19]. However, almost all of these studies involved the use of a mesh seeded with stem cells, in which the effect of stem cells could not be directly evaluated due to the effect of the mesh on the host tissue. Additionally, the use of mesh greatly increases the cost of POP treatment. In the pelvic floor dysfunction field, there have been many scientific and clinical studies on the treatment of stress urinary incontinence (SUI) by the periurethral injection of stem cells. Inspired by the periurethral injection of stem cells for SUI, we directly injected MSCs into the vaginal wall in this study.

Rhesus macaques are considered the best animal for modeling and studying POP, given their similarity to humans in terms of pelvic floor anatomy and histological structure. In addition, rhesus macaques are intermittently bipedal and give birth to infants with a relatively large head diameter, which may contribute to the development of POP. Since menopause and pregnancy are considered major risk factors for POP [9], we selected multiparous rhesus macaques and performed bilateral ovariectomy 3 months before the MSC injection to induce a menopausal status.

In this study, we used rhesus macaques to establish the animal model and directly injected MSCs into the vaginal wall. The aim of this study was to evaluate the impact of MSCs on vaginal repair in a bilaterally ovariectomized rhesus macaque model. MSC transplantation effectively repaired and normalized the fibromuscular structures of the vagina, indicating the potential of this approach as a treatment for POP.

## Methods

### Isolation and culture of MSCs

The human umbilical cord was obtained from a healthy and full-term birth with informed consent from the donor, and the procedures were approved by the Ethics Committee of Peking Union Medical College Hospital (JS-2043). MSCs were isolated according to a previously described method [20]. Briefly, the cord was rinsed with ice-cold phosphate-buffered saline to remove blood clots. Wharton’s jelly around the cord vessels was isolated and dissected into 1- to 2-mm pieces. After partial digestion of the tissue pieces in trypsin solution, the pieces were placed on tissue culture dishes in MSC medium (Viraltherapy Technologies, Wuhan, China) and incubated at 37 °C in 5% CO_2_. The cells (passage 0) grew out from the adherent explants over approximately 7 to 10 days of culture. MSCs from passages 3–6 were used in the following experiments.

### Animals and surgical procedures

Ten female multiparous rhesus macaques, aged 10 years, were provided by the Beijing Xieerxin Institute of Biological Resources. Experimental protocols were approved by the Ethics Committee of Beijing Xieerxin Institute of Biological Resources (E20190401). All animals were kept in single cages according to the current national animal welfare standards. Routine laboratory tests and regular examinations by veterinarians during a quarantine period were used to certify that these experimental animals were pathogen-free and in good physical condition. Animals received water ad libitum and scheduled chow supplemented with fresh fruit, vegetables, and multiple vitamins daily. Animals were raised under standard laboratory conditions (temperature, 20–22°C; relative humidity, 50–70%; 12 h/12 h light/dark cycle).

All rhesus macaques underwent bilateral ovariectomy to induce a menopausal status. Compound ketamine was used for intramuscular anesthesia at a dosage of 1–2 mg/kg. Then, a ventral midline incision in the upper abdomen was made under sterile conditions, and the ovaries were well exposed and excised. All animals were administered penicillin to prevent infection after the operation.

Ten bilaterally ovariectomized rhesus macaques were divided into two groups (n=5/group): the saline group and the MSC group. Three months after ovariectomy, (1) animals in the control group received vaginal subepithelial injections of 1.8 mL saline, (2) while animals in the MSC group received injections of 1×10^8^ MSCs (in 1.8 mL of saline) at the same sites. The vaginal length of the rhesus macaques was approximately 5 cm. Injections were performed at six points of the vaginal wall: two points on the anterior vaginal wall, two points on the posterior vaginal wall, one point on the left vaginal wall, and one point on the right vaginal wall. Specifically, a straight clamp was used to dilate and expose the vaginal wall. The two injection points on the anterior vaginal wall were 1.5 cm and 3.5 cm above the vaginal introitus on the midline of the anterior vaginal wall. Similarly, the two points on the posterior vaginal wall were 1.5 cm and 3.5 cm above the introitus on the midline of the posterior vaginal wall. The injection points on the left and right vaginal wall were located 2.5 cm above the vaginal introitus. At 12 weeks after injection, the rhesus macaques were sacrificed, and the vagina was harvested for the evaluation of histomorphology and biomechanical properties.

After anesthesia was induced, we opened the abdominal cavity to free the vagina to the vaginal orifice and disarticulated the pubic symphysis to isolate the vaginal orifice from the surrounding perineal skin. Then, the vaginal tube was harvested intact. After isolating the vagina, the animal was euthanized. The collected vagina was divided into the anterior and posterior vaginal walls. The posterior vaginal wall was cut transversely into proximal and distal segments. The proximal segment was immediately immersed in 10% neutral-buffered formalin for histological evaluation. The distal segment was stored in liquid nitrogen for the molecular study. The anterior vaginal wall was wrapped in saline gauze moistened with 0.9% normal saline and placed in a refrigerated box (built-in ice box in which the temperature can be maintained at 0–4°C).

### Masson trichrome staining, sirius red staining, and Verhoeff-van Gieson staining

The vaginal tissue was fixed in 10% formalin for 24 h, embedded in paraffin, and cut into 5-μm-thick sections. The vaginal tissue sections were stained following the standard procedures for Masson trichrome staining, sirius red staining, and Verhoeff-van Gieson staining. For image analysis, the slides were viewed under a Nikon Eclipse CI microscope, and images of five randomly selected fields per slide were captured with a Nikon DS-U3 camera. Image-Pro Plus computer software was used to calculate the percentage of collagen I and collagen III (sirius red stain, ×200 magnification). The percentage of elastin in the lamina propria was analyzed using National Institutes of Health ImageJ software (Verhoeff-van Gieson stain, ×400 magnification).

### Immunohistochemistry

Immunohistochemistry was performed as previously described using anti-α-smooth muscle actin (α-SMA) antibody (ab5694, Abcam) to identify smooth muscle in the vaginal tissue [21]. Five images per slide of the muscularis region (×200 magnification) were captured under a Nikon Eclipse CI microscope with a Nikon DS-U3 camera. Vascular smooth muscle was excluded manually from each image, and the fraction of smooth muscle was determined by computing the area of α-actin staining relative to the total area of nonvascular muscularis using National Institutes of Health ImageJ software.

### Immunofluorescence

von Willebrand factor (vWF), a glycoprotein produced by endothelial cells, is routinely used to identify vessels in tissue sections. Immunofluorescence staining was performed with a mouse monoclonal primary antibody against vWF (ab201336, Abcam). Five images per slide (×200 magnification) of the lamina propria region were captured under a Nikon Eclipse CI microscope with a Nikon DS-U3 camera. The results are expressed as the mean number of microvessels per high-power field.

### RT-qPCR

Quantitative real-time polymerase chain reaction (RT-qPCR) was used to determine the relative levels of mRNA in vaginal tissues. Total RNA was extracted using TRIzol reagent (Invitrogen, USA) and reverse transcribed using HiScript II Q RT SuperMix for qPCR (R223-01, Vazyme, China) according to the manufacturer’s instructions. RT-qPCR was performed using an Applied Biosystems QuantStudio system with ChamQ SYBR Green qPCR Master Mix (Q331-02, Vazyme, China). The Delta-Delta-Ct (ddCt) method was used to determine relative gene expression. The relative expression of mRNA was normalized to that of the housekeeping gene glyceraldehyde-3-phosphate dehydrogenase (GAPDH).

### Biomechanical testing

Uniaxial tensile biomechanical testing was performed within 24h after obtaining the specimen. The load (Newtons) and elongation (millimeters) were recorded to generate a load-elongation curve. Three parameters describing the biomechanical properties of the vaginal wall were obtained: ultimate load (N), ultimate strain, and elastic modulus (MPa). Ultimate load defines the point of tissue disruption on the load-elongation curve. Strain is the percent change in the length of the material. Ultimate strain is maximal elongation divided by the original length. Stress is the measured load divided by the cross-sectional area. Elastic modulus is the ratio of stress to the corresponding strain in the linear region of stress-strain curve. It is the measure of stiffness of a material. In terms of the stress-strain curve, elastic modulus is the slope of the stress-strain curve in the range of linear proportionality of stress to strain.

### Statistics

Kolmogorov–Smirnov test was used to analyze whether the data were normally distributed. Following confirmation of normal distribution, unpaired Student’s t test or Mann-Whitney was carried out for checking differences between both groups. The data are presented as mean ± standard deviation (SD). P values <0.05 was considered statistically significant. All statistical analyses were performed using SPSS version 26.0 (IBM Corp, Armonk, NY).

## Results

### Isolation and characterization of MSCs

A schematic of the MSC isolation and characterization protocol is shown in Supplementary Figure 1. Flow cytometric analysis showed positive expression of CD90, CD105, CD73, CD29, and CD44 and low expression of CD34 and CD45, indicating that the cells isolated from human umbilical cord had MSC characteristics.

### Demographics of rhesus macaques

Demographic data from each group are illustrated in Table 1. All animals have similar age, parity, and BMI.

**Table 1:** Demographics of rhesus macaques in the study

Groups	Age (years)	Parity	CRL (cm)	Weight (kg)	BMI (kg/m^2^)
Saline	10.4±0.6	4 (3, 4)	58.4±4.4	8.7±1.3	25.5±0.9
MSC	10.2±0.4	4 (3, 4)	58.0±6.8	8.9±1.5	26.7±4.1
*P* value	0.545	0.820	0.915	0.838	0.532

Age, CRL, weight, and BMI are expressed as the mean ± standard deviation. Parity is expressed as median (interquartile range). *BMI*, body mass index; *CRL*, crown-rump length

### Effect of MSCs on vaginal histomorphology

**Fig. 1:**
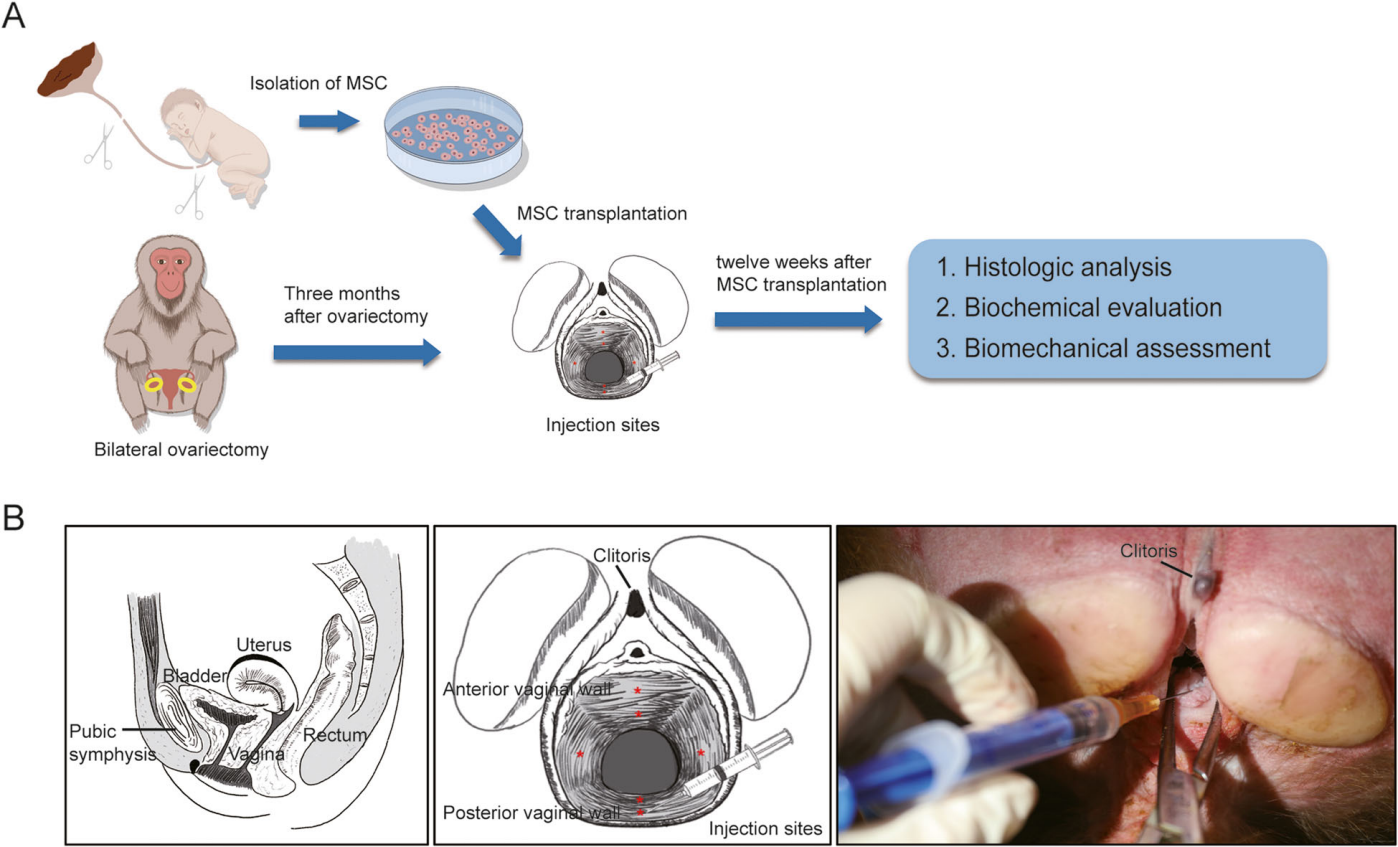
Graphic abstract and MSC injection sites. **A** Schematic of animal model establishment, cell isolation, transplantation, and downstream analysis. **B** Schematic of pelvic anatomical structure and MSC injection sites of rehsus macaque

MSCs were injected into vaginal subepithelial sites of the ovariectomized rhesus macaque and the effects were evaluated 12 weeks after transplantation (Fig. 1A). The six specific vaginal wall injection sites in the rhesus macaques are shown in Fig. 1B. All animals had a normal recovery after the injections.

**Fig. 2:**
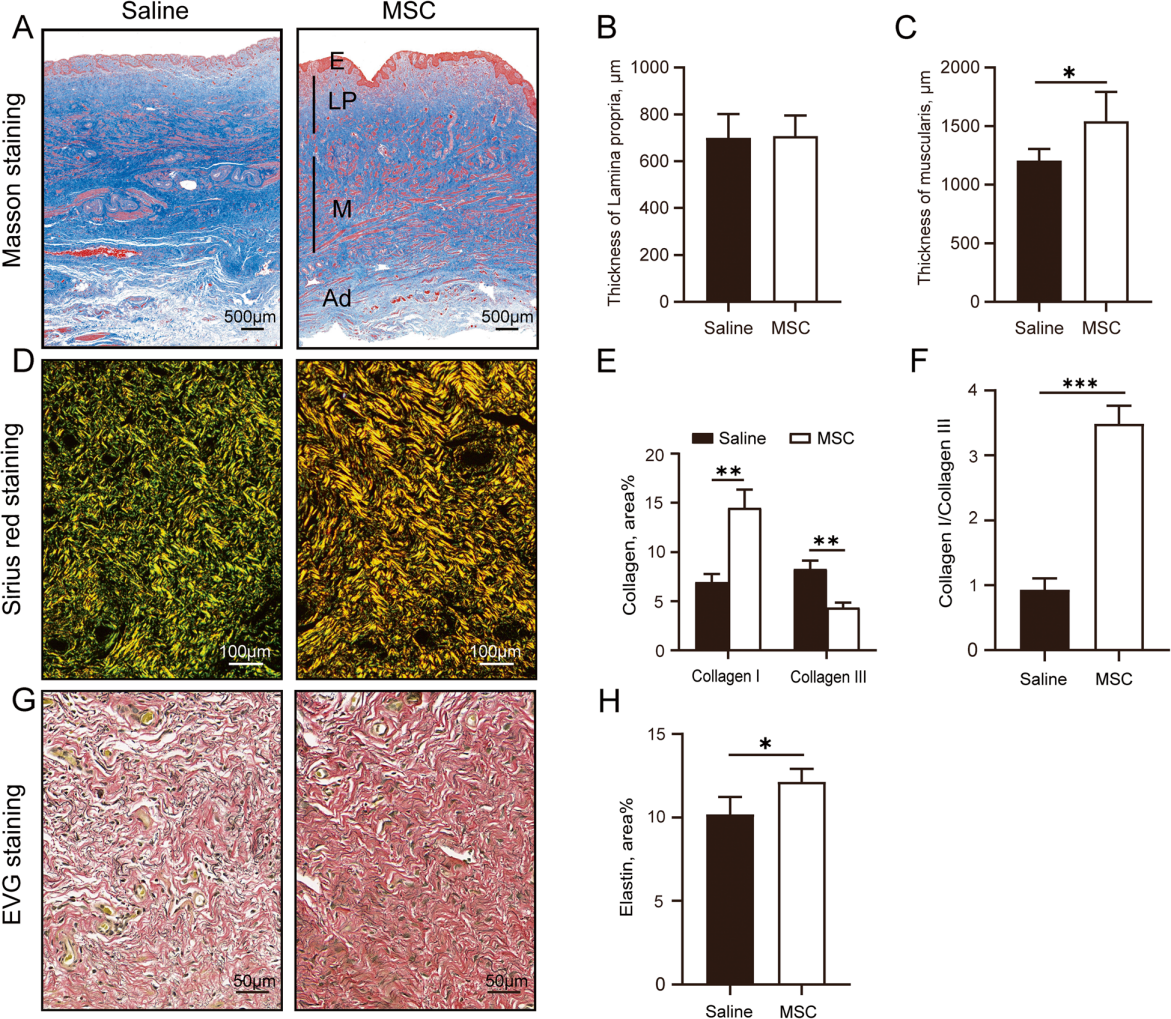
Histological staining and changes of collagen and elastin content in the vagina. **A** Masson trichrome staining of full-thickness vaginal tissue (×50). E, epithelium; LP, lamina propria; M, muscularis; Ad, adventitia. **B** Quantification of the thickness of lamina propria. **C** Quantification of the thickness of muscularis. **D** Birefringence images of Sirius red staining of lamina propria layer (×200). **E** Quantitative analysis of the percentage of collagen I and collagen III. **F** Quantitative analysis of the ratio of collagen I/collagen III. **G** Verhoeff-van Gieson staining of lamina propria layer (×400). Elastic fibers and nucleus were stained black. **H** Quantitative analysis of the percentage of elastin. Data were presented as the mean ± standard deviation. n=5 animals/group. *p ≤ 0.05, **p ≤ 0.01, ***p <0.001

To examine the morphology of the vaginal wall, Masson trichrome staining was applied (Fig. 2A). Similar to humans, the vaginal wall of rhesus macaques comprises the following layers: the epithelium (stained pink), lamina propria (stained blue), muscularis (stained pink), and adventitia (stained blue). There was no significant difference in the thickness of the lamina propria between the saline group and the MSC group (Fig. 2B). However, there was a significant increase in the thickness of the muscularis layer of the vagina in the MSC group compared with the saline injection group (Fig. 2C).

### Changes in the collagen composition in the vagina

Sirius red birefringence was used to assess the collagen in the lamina propria layer of the rhesus macaque vagina (Fig. 2D). The slides were viewed under a light microscope equipped with a polarizing filter to identify the birefringent sirius red-stained collagen fibers. Under polarized microscopy, the collagen fibers showed a mixed proportion of birefringent staining patterns ranging from green to orange/red; orange/red indicated collagen I, while green indicated collagen III. At 12 weeks postinjection, quantitative analysis showed that the percent area of collagen I in the lamina propria was significantly increased and that the percent area of collagen III was significantly decreased (Fig. 2E) in the MSC group. Accordingly, the MSC group showed a significantly higher collagen I/III ratio (Fig. 2F) than the saline group 12 weeks after injection.

### Increased elastic fiber content in the vagina

Verhoeff-van Gieson staining was used to assess the percent area of elastic fibers in the lamina propria of the vaginal segment (Fig. 2G). In the stained sections, elastin appeared black, collagen appeared red, and smooth muscle appeared yellow. In the MSC group, elastin accounted for 12.13% of the lamina propria, which was significantly higher than the corresponding value of 10.19% in the saline group (Fig. 2H).

### Increased smooth muscle content in the vagina

**Fig. 3:**
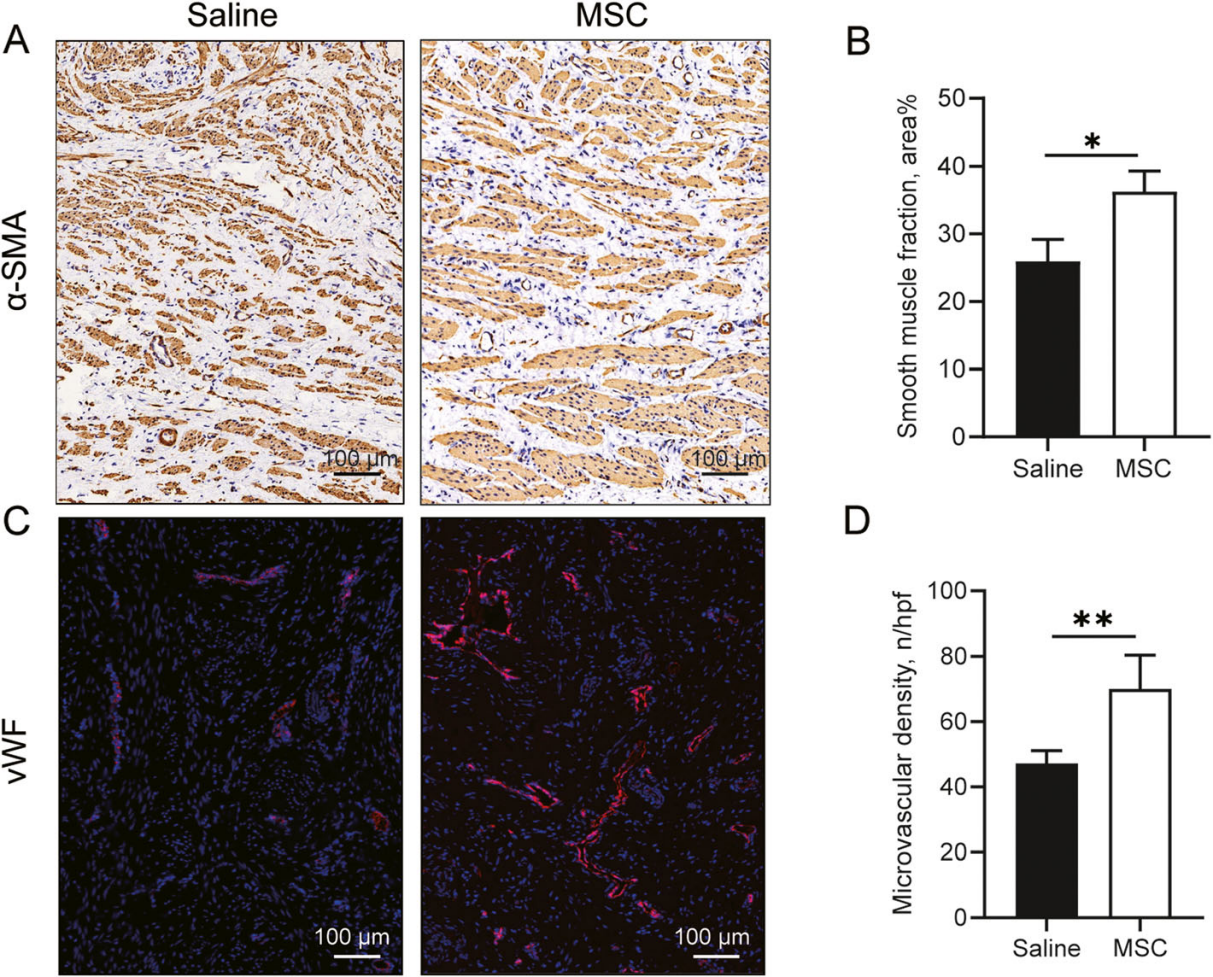
Effect of MSCs on the smooth muscle and microvascular density of the vaginal tissue. **A** Immunohistochemistry of α-SMA for morphology and quantity of smooth muscle (×200). **B** Quantitative analysis of α-SMA staining. **C** Immunofluorescence staining of von Willebrand factor (vWF) for evaluating microvascular density (×200). **D** Quantification of microvascular density. The number of microvessels per high power field (hpf) under a light microscope. Data were presented as the mean ± standard deviation. n=5 animals/group. *p ≤ 0.05, **p ≤ 0.01.

Immunohistochemistry was used to assess the morphology and quantity of the nonvascular smooth muscle in the muscularis layer of the rhesus macaque vaginal wall (Fig. 3A). Compared with those in the MSC group, the smooth muscle bundles in the saline group appeared to be disorganized and smaller. In addition, the fraction of smooth muscle in the nonvascular muscularis layer was 36.26% in the MSC group, which was significantly higher than the 25.95% in the saline group (Fig. 3B).

### Increased microvascular density in the vagina

Immunofluorescence was used to determine the microvascular density in the lamina propria of vaginal tissue by staining with von Willebrand factor (vWF) (Fig. 3C). We examined the vessels under a light microscope and counted the number of vessels per high-power field (×200 magnification). Significantly more vWF-positive vessel profiles were observed in the MSC group than in the saline group (Fig. 3D).

### Effect of MSCs on gene expression

**Fig. 4:**
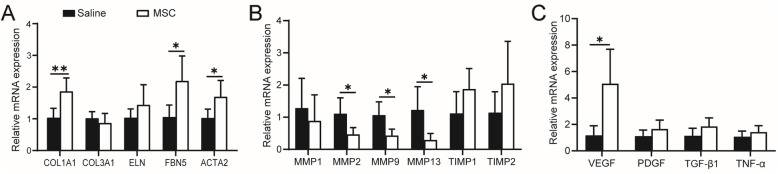
Gene expression of the vaginal tissue by RT-qPCR. **A** Genes related to extracellular matrix (ECM) and smooth muscle. **B** Genes related to ECM remodeling. **C** Genes related to growth factors and inflammation factors. Data were presented as the mean ± standard deviation. n=5 animals/group. *p ≤ 0.05, **p ≤ 0.01.

RT-qPCR was used to analyze the expression of smooth muscle (*ACTA2*), extracellular matrix (ECM)-associated genes (collagen I α I (*COL1A1*), collagen III α 1 (*COL3A1*), elastin (*ELN*), fibulin-5 (*FBN5*)), and genes involved in ECM remodeling (matrix metalloproteinases (MMPs), tissue inhibitor of metalloproteinases (TIMPs)) 12 weeks after injection. At the mRNA level, the MSC group showed significantly upregulated smooth muscle, collagen I, fibulin-5 expression, and downregulated MMP2, MMP9, and MMP13 expression (Fig. 4A, B). In addition, we quantified the mRNA levels of vascular endothelial growth factor (*VEGF*), transforming growth factor β1 (*TGF-β1*), tumor necrosis factor α (*TNF-α*), and platelet-derived growth factor (*PDGF*). At the mRNA level, the MSC group significantly upregulated *VEGF* expression (Fig. 4C). The primer sequences used for RT-qPCR are described in Supplementary Table 1.

### Effect of MSCs on the biomechanical properties of the vaginal tissue

**Fig. 5:**
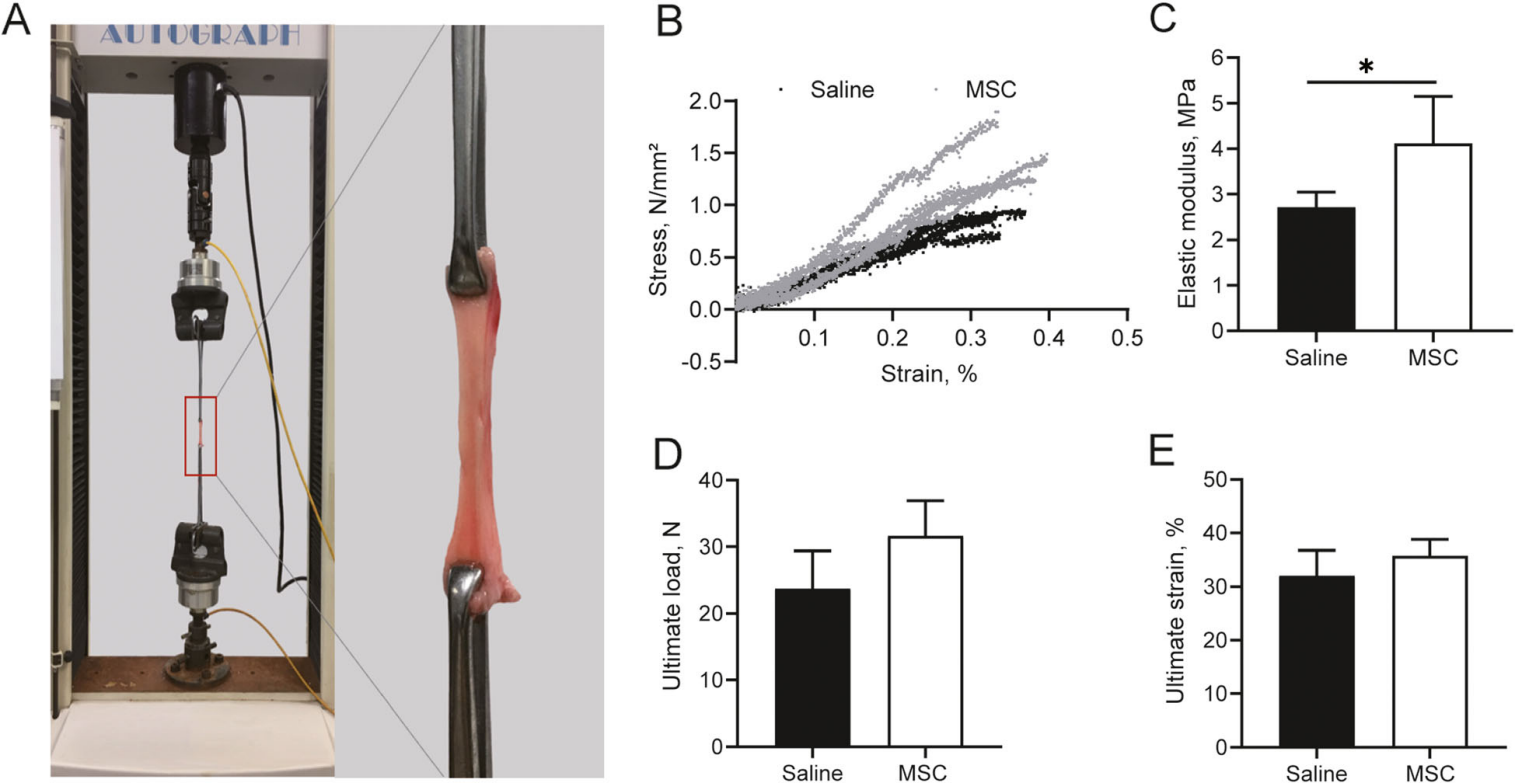
Biomechanical properties of vaginal tissue. **A** Demonstration process of biomechanical testing. **B** Stress-strain curves in the elastic regime of vaginal tissue. **C** Comparison of elastic modulus, **D** ultimate load, and **E** ultimate strain between the two groups. n=5 animals/group. *p ≤ 0.05

The biomechanical properties of the vaginal tissue were assessed using uniaxial biomechanical testing. The generated load-elongation curves were nonlinear with the characteristic toe, linear, and failure regions. Figure 5B shows the stress-strain curves of vaginal tissue in the “elastic phase.” There was no significant difference in the ultimate load or ultimate strain at failure between the MSC group and the saline group at 12 weeks after injection (Fig. 5D, E). The elastic modulus in the MSC group was significantly higher than that in the saline group (Fig. 5C, 4.12±1.04 versus 2.71±0.34 MPa), indicating that the vaginal tissue injected with MSCs was stiffer than that not injected with MSCs.

## Discussion

At present, surgical treatments for POP do not repair weak vaginal tissue and just provide support, and these treatments may be accompanied by severe complications. Stem cell-based regenerative therapy, due to its ability to repair and restore damaged tissue, may have potential as a treatment strategy for POP. In this study, we demonstrated that the injection of MSCs could repair the vaginal tissue of rhesus macaques after bilateral ovariectomy through remodeling of the ECM and the formation of smooth muscle and microvessels, suggesting the potential of MSCs to facilitate the reconstruction of weak vaginal tissue as a new prospective treatment for POP.

Structural defects in vaginal tissue are believed to be closely related to the occurrence and development of POP [22, 23]. The lamina propria and muscularis are the two important layers that maintain the supportive functions of the vaginal wall. The clinical manifestation of POP may occur due to the dysregulation of ECM metabolism [24]. Collagen and elastin are fundamental components of the ECM that provide support for the pelvic floor [25]. Previous studies have found reduced collagen I expression and a reduced collagen I/III ratio as well as increased of III expression in vaginal tissue in POP [26, 27]. The strong association between POP and changes in collagen suggests that collagen should be seriously considered in the design of therapeutic strategies for POP. In our study, the MSC group exhibited a significant increase in collagen I expression and the ratio of collagen I/III compared with the saline group. Our results indicate gradual new tissue growth promoted by the MSC treatment in contrast to the continual decrease in collagen after menopause. Elastin is also an important component of the vaginal wall, and fibulin-5 plays a vital role in elastin formation by guiding the assembly of elastic fibers [28]. Previous studies have shown abnormal elastic fiber formation and spontaneous POP development after parturition in fibulin-5 knockout mice [29, 30]. In our study, the content of elastin and fibulin-5 was significantly increased in rhesus macaques treated with MSCs at 12 weeks after injection. Similarly, Lin et al. reported that a significantly higher elastin content in the MSC group than in the control group in the treatment of SUI [31], suggesting that MSCs might promote elastin production.

The vaginal ECM is under constant remodeling, and the balance between MMP and TIMP expression is critical for ECM homeostasis. Previous studies have shown an association between increased MMP/decreased TIMP expression and the occurrence of POP [32, 33]. Additionally, MSCs have been shown to secrete ECM-mediating factors, including MMPs and TIMPs [34]. We assessed the expression of MMP1, MMP2, MMP9, and MMP13, which can specifically cleave collagen I, collagen III, and elastin [35], and the expression of their inhibitors, TIMP1 and TIMP2. In our study, the mRNA expression levels of MMP2, MMP9, and MMP13 were significantly decreased in the MSC group compared with the saline group. However, statistical analysis revealed no significant difference in the expression of MMP1, TIMP1, or TIMP2 between the two groups. We infer that the downregulation of MMP expression in the MSC group may play a role in increasing the collagen and elastin content by reducing degradation.

A higher microvascular density in the lamina propria layer of the vaginal tissue in the MSC group was expected, since MSCs are known to secrete angiogenic factors, such as VEGF [36, 37]. VEGF is a classic growth factor that induces neovascularization by promoting the migration and proliferation of microvascular endothelial cells. In our study, histological analysis showed increased microvascular density in the MSC group. In addition, the mRNA expression of VEGF was significantly increased in the MSC group compared with the saline group. In line with our research results, others have also demonstrated that MSCs promote neovascularization by secreting VEGF [38–40]. Neovascularization is also an important aspect of healthy tissue regeneration that merits adequate attention in the design of POP treatment methods.

Previous studies have shown that alterations in smooth muscle morphology and function in the vaginal tissue may participate in the pathogenesis of POP [41]. The fractional area of nonvascular vaginal smooth muscle in the muscularis of women with POP was significantly decreased compared with that of women without POP [42, 43], suggesting that the increase in vaginal smooth muscle may be of critical importance for POP treatment. Our results demonstrated that MSCs promoted the formation of the smooth muscle. Consistent with our results, MSC therapies have been shown to increase the content of smooth muscle in the treatment of SUI in a rat model [44, 45]. MSCs have the potential to differentiate into functional smooth muscle cells [46]. However, De Coppi et al. reported that MSCs mainly regulated smooth muscle via a paracrine mechanism and that their effect via direct differentiation was limited [47]. Further research is still needed to determine whether the increased smooth muscle in our study was derived from the differentiation of MSCs or their secretion of factors to promote autologous tissue regeneration.

POP can change the biomechanical properties of the vaginal wall. Epstein et al. reported that the vaginal wall of women with POP was significantly more extensible than that of women without POP and that increased vaginal extensibility was associated with increased POP severity [11]. These findings appear to be in agreement with those of previous histological studies of vaginal tissue showing less collagen I and more collagen III in women with than without POP, as collagen I provides tensile strength and stiffness and collagen III affects tissue extensibility and elasticity [48]. Our results showed that the elastic modulus was significantly higher in the MSC group, indicating that the vaginal tissue injected with MSCs was stiffer than that not injected with MSCs. In agreement with our findings, Zou et al. reported that MSCs promoted the elastic modulus in the treatment of SUI in a rat model [49]. The changes in biomechanical properties following MSC injection in our study can be explained by changes in ECM deposition and smooth muscle regeneration.

There are some limitations to our study. A key limitation is the POP animal model. The changes in vaginal tissue in ovariectomized animals may not entirely reflect the changes in the vaginal tissue caused by POP. However, there is currently no effective method to establish a POP animal model. Lacks of cell fate tracking after MSC transplantation and the functional mechanism of MSC therapy are also important limitations of this study, which will be conducted in our future study. The small sample size is another limitation of the present study. To our knowledge, this is the first study to evaluate stem cell therapy for the repair of the weak vaginal tissue in rhesus macaques after bilateral ovariectomy without a traditional mesh, and treatment with MSCs showed a positive effect on vaginal tissue repair. This study demonstrates a new potential therapeutic approach for POP and provides a basis for preclinical research on the clinical application of stem cell therapies.

## Conclusion

In conclusion, the vaginal transplantation of MSCs could repair the weak vaginal tissue in bilateral ovariectomized rhesus macaques by promoting ECM ingrowth, neovascularization, and smooth muscle formation and improve the biomechanical properties of the vagina by enhancing the elastic modulus. The potential of MSCs in the repair of the weak vaginal tissue offers a new prospective treatment for POP.

## Supplementary information

**Additional file 1:** Supplementary Figure 1. Isolation and characterization of MSCs. (A) Schematic of isolation of mesenchymal stem cells (MSCs) from human umbilical cord. (B) Morphology of MSCs under light microscope. (C) Immunophenotype of MSCs by flow cytometry.

**Additional file 2:** Supplementary Table 1. Primers used for PCR amplification

## Data Availability

All data generated or analyzed during this study are included in this published article and its supplementary information files.

## Abbreviations

COL1A1: Collagen I α I
COL3A1: Collagen III α 1
ECM: Extracellular matrix
ELN: Elastin
FBN5: Fibulin-5
GAPDH: Glyceraldehyde-3-phosphate dehydrogenase
MMP: Matrix metalloproteinase
MSC: Mesenchymal stem cell
PDGF: Platelet-derived growth factor
POP: Pelvic organ prolapse
RT-qPCR: Quantitative real-time polymerase chain reaction
SD: Standard deviation
SMA: Smooth muscle actin
SUI: Stress urinary incontinence
TGF-β1: Transforming growth factor β1
TIMP: Tissue inhibitor of metalloproteinase
TNF-α: Tumor necrosis factor α
VEGF: Vascular endothelial growth factor
vWF: von Willebrand factor
