# Supplementary figures and images for: Mesenchymal stem cell transplantation for vaginal repair in an ovariectomized rhesus macaque model
Source: Stem Cell Res Ther. 2021 Jul 15;12:406. doi: 10.1186/s13287-021-02488-2 (PMC8281669; doi:10.1186/s13287-021-02488-2)

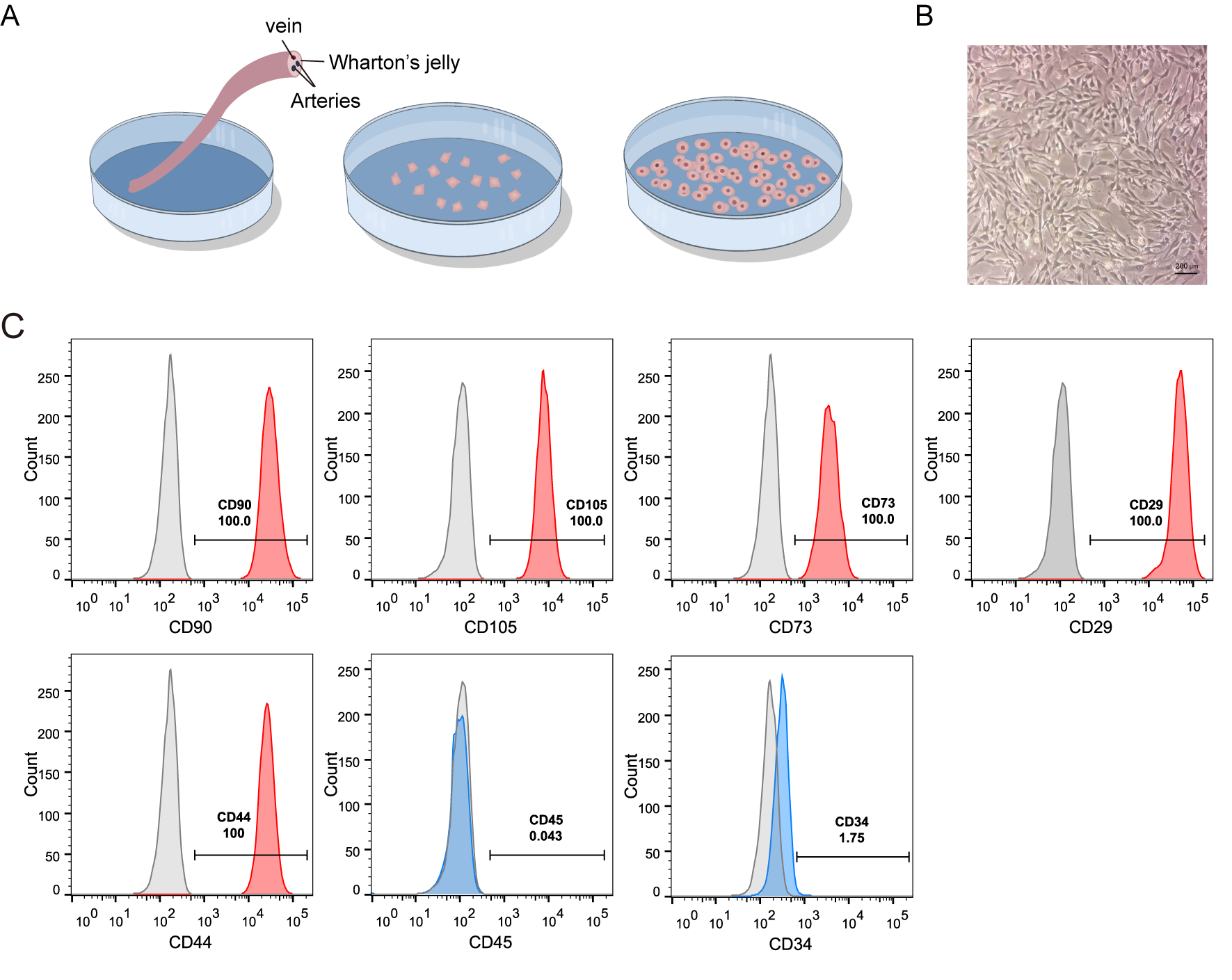

Supplement: Supplementary file 1 — Additional file 1: Supplementary Figure 1. Isolation and characterization of MSCs. (A) Schematic of isolation of mesenchymal stem cells (MSCs) from human umbilical cord. (B) Morphology of MSCs under light microscope. (C) Immunophenotype of MSCs by flow cytometry. [file 13287_2021_2488_MOESM1_ESM.tif]
