# Supplementary material for: Mesenchymal stem cell transplantation for vaginal repair in an ovariectomized rhesus macaque model
Source: Stem Cell Res Ther. 2021 Jul 15;12:406. doi: 10.1186/s13287-021-02488-2 (PMC8281669; doi:10.1186/s13287-021-02488-2)
Supplement: Supplementary file 2 — Additional file 2: Supplementary Table 1. Primers used for PCR amplification [file 13287_2021_2488_MOESM2_ESM.docx]

**Figure legend**

**Supplementary Figure 1. Isolation and characterization of MSCs.** (A) Schematic of isolation of mesenchymal stem cells (MSCs) from human umbilical cord. (B) Morphology of MSCs under light microscope. (C) Immunophenotype of MSCs by flow cytometry.

**Supplementary Table 1. Primers used for PCR amplification**

| Genes | Forward | Reverse |
| --- | --- | --- |
| *GAPDH* | AAGGTGAAGGTCGGAGTCAAC | ACTTGCCATGGGTGGAATCAT |
| *COL1A1* | TGACGAGACCAAGAACTGCC | CAGGAGATTACCTCGACGCC |
| *COL3A1* | TGAATCATGCCCCACTGGTC | TCGTCCGGGTCTACCTGATT |
| *ELN* | TCAAAGCTGGATTCGCCCAT | CCACAAGAGCAAGGTGGCTA |
| *FBN5* | GCGCTGTAATCTGAACCAGC | GACTGGCGATCCAGGTCAAA |
| *ACTA2* | CGTGCTGGACTCTGGAGATG | CGCTCAGCGGTAGTAACGAA |
| *MMP1* | ACATGCGCACAAATCCCTTC | TCGGCAAATTCGTAAGCAGC |
| *MMP2* | TGTCCACTGTTGGTGGGAAC | TCGCACACCACATCTTTCCA |
| *MMP9* | AGCGAGGTAGACCGGATGTT | AACTCACGCGCCAGTAGAAG |
| *MMP13* | CAAGATGCGGGGTTCCTGAT | TCGCCATGCTCCTTAGTTCC |
| *TIMP1* | GGACGGACTCTTGCACATCA | TGTGCATTCCTCACAGCCAA |
| *TIMP2* | GGCGTTTTGCAATGCAGATG | CTCAGGCCCTTTGAACATCTTT |
| *VEGF* | TAAGTCCTGGAGCGTTCCCT | CCCAAAGCACAGCAATGTCC |
| *TGF-β* | GCCTCCCGCAAATACTTTTCC | GCTGTATTTCTGGTACAGCTCC |
| *TNF-α* | TCTTCTCCTTCCTGCTCGTG | AGTCGAGATAGTCGGGCAGA |
| *PDGF* | ACCGTAGTCAGGGTCCAACT | GGGACAGCTTCCTCAATGCT |
